# Changes to human sleep architecture during long‐duration spaceflight

**DOI:** 10.1111/jsr.14345

**Published:** 2024-11-10

**Authors:** Oliver Piltch, Erin E. Flynn‐Evans, Millennia Young, Robert Stickgold

**Affiliations:** ^1^ Division of Life Sciences, Department of Neuroscience Harvard University Cambridge Massachusetts USA; ^2^ Vagelos College of Physicians & Surgeons Columbia University New York New York USA; ^3^ Fatigue Countermeasures Laboratory, Human Systems Integration Division NASA Ames Research Center Moffett Field California USA; ^4^ Biomedical Research and Environmental Sciences Division Human Health and Performance Directorate, NASA Johnson Space Center Houston Texas USA; ^5^ Center for Sleep and Cognition, Department of Psychiatry Beth Israel Deaconess Medical Center and Harvard Medical School Boston Massachusetts USA

**Keywords:** astronaut, non‐rapid eye movement, rapid eye movement, sleep, sleep architecture, spaceflight

## Abstract

Both rapid eye movement and non‐rapid eye movement sleep are important for cognitive function and well‐being, yet few studies have examined whether human sleep architecture is affected by long‐duration spaceflight. We recorded 256 nights of sleep from five crew members before (*n* = 112 nights), during (*n* = 83 nights) and after (*n* = 61 nights) ~6‐month missions aboard the Mir space station, using the Nightcap sleep monitor. We compared sleep outcomes (including total sleep time, efficiency, latency, rapid eye movement and non‐rapid eye movement) during spaceflight with those on Earth. We also evaluated longitudinal changes over time in space. We found that wakefulness increased by 1 hr in space compared with on Earth. Over time in space, rapid eye movement was initially reduced and then recovered to near preflight levels at the expense of non‐rapid eye movement sleep. Upon return to Earth, sleep architecture returned to preflight distribution. Our findings suggest that spaceflight may alter sleep architecture and should be explored further.

## 1 INTRODUCTION

Normal human sleep follows predictable changes in sleep stages over the course of a night among healthy, non‐shift‐working individuals (Dijk & Czeisler, 1995). Sleep is regulated by the build‐up of homeostatic sleep pressure and the circadian rhythm, which promotes wakefulness during the day and allows for consolidated sleep at night (Borbely, 1982). This diurnal sleep architecture evolved on Earth where the 24‐hr rotation of the planet provides daily light–dark cycles that entrain the circadian system and facilitate predictable sleep at night. Each stage of sleep contributes to different aspects of cognitive function, such as learning and memory consolidation (Stickgold, 2005), rehearsal of motor skills, and emotional regulation (Vandekerckhove & Wang, 2018), while functions such as vigilant attention (Hudson et al., 2020) deteriorate with overall sleep loss, making quality sleep critical for adequate cognitive performance in safety‐sensitive operations.

Humans sleep significantly less in space compared with on Earth (Barger et al., 2014; Dijk et al., 2001; Frost Jr. et al., 1976; Gundel et al., 1997; Monk et al., 2001; Santy et al., 1988). This may be due, in part, to the circadian misalignment arising from the loss of the 24‐hr solar light–dark cycle on Earth or inappropriate scheduling (Flynn‐Evans, Barger, et al., 2016), poor sleep environment conditions (Caddick et al., 2017), and workloads that elevate stress and prevent adequate sleep opportunity (Flynn‐Evans, Gregory, et al., 2016). It is unclear whether microgravity directly influences sleep duration and/or staging, and if the observed reduction in sleep duration results from the specific loss of one sleep stage or a non‐specific reduction in both rapid eye movement (REM) and non‐REM (NREM) sleep. It is difficult to assess sleep architecture in space because agencies such as NASA impose restrictions on the equipment that can be flown based on research priorities, flight certification requirements, and payload weight restrictions. The few studies that have examined sleep architecture in space have been mixed, with one study showing increased REM sleep duration during spaceflight (Frost Jr. et al., 1976), three demonstrating shorter REM latency (Frost Jr. et al., 1976; Gundel et al., 1993; Gundel et al., 1997), two presenting increased slow‐wave sleep (SWS; Frost Jr. et al., 1976; Monk et al., 1998), and two reporting decreased SWS (Dijk et al., 2001; Monk et al., 1998). Notably, only two of these studies involved data collection from long‐duration missions (> 30 days), but their data were presented as four isolated case studies (Frost Jr. et al., 1976; Gundel et al., 1997). Similarly, most of these studies collected very little data in space, with four studies reporting collection of 4–7 nights of data from 1‐5 participants (Dijk et al., 2001; Gundel et al., 1993; Gundel et al., 1997; Monk et al., 1998), while one study reported collection of 12, 18 and 20 nights from three individual crewmembers on Skylab (Frost Jr. et al., 1976). The diversity of these studies involving different missions, schedules and environmental conditions makes it difficult to disentangle whether the observed changes were due to individual or mission factors or due to the influence of microgravity itself. In addition, most of the participants in prior studies spent less than 2 months in space, making it unclear whether the reported changes in sleep staging were transient or stable.

The purpose of the present study was to characterize global changes in sleep architecture over multiple days in space compared with on Earth using a minimally intrusive, validated device designed to distinguish between REM, NREM and wakefulness. A further aim was to assess sleep architecture over the course of a long‐duration mission to identify any longitudinal changes.

## 2 METHODS

### 2.1 Participants

**FIGURE 1:**
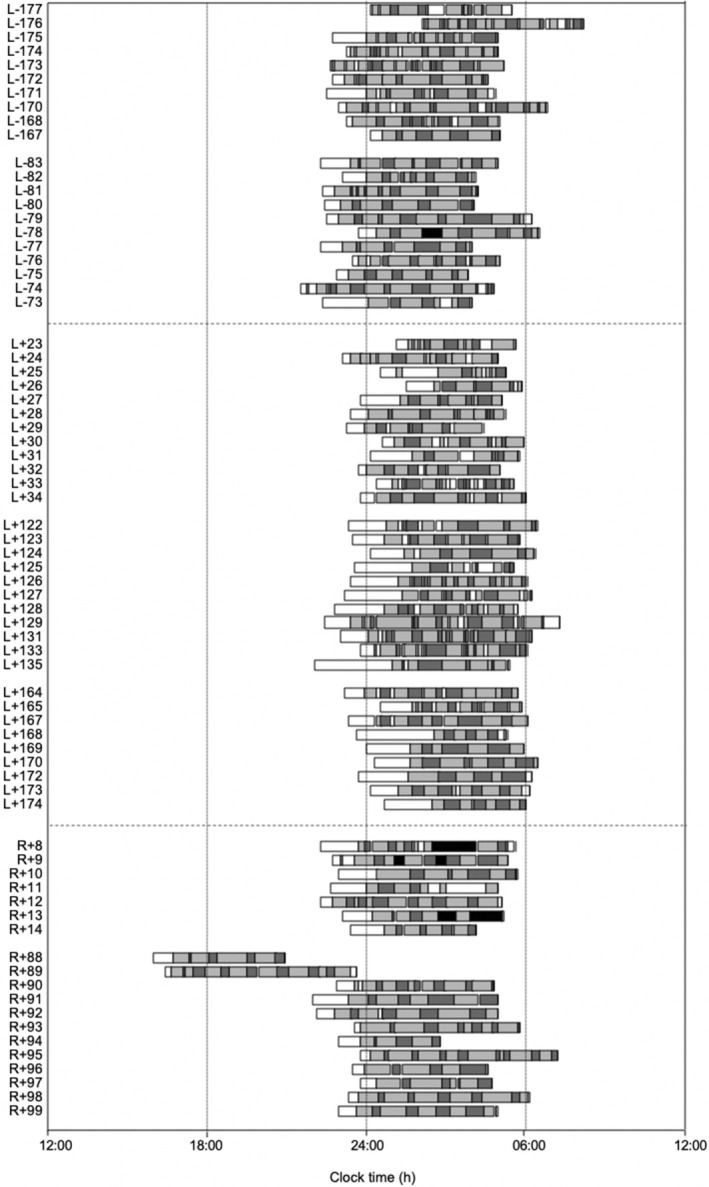
Raster plot from one study participant. Numbers preceded by L− represent the night of preflight data collection relative to launch (L = launch). Numbers preceded by L+ represent inflight data collection nights after launch. Numbers preceded by R+ represent data collection during the postflight phase relative to return (R = return). Boxes represent sleep opportunity, white = in bed awake, dark grey = rapid eye movement (REM) sleep, light grey = non‐REM sleep (NREM), black = unscorable.

Participants were US astronauts and Russian cosmonauts selected for missions aboard the space station Mir. There were no other exclusionary criteria. Participant behaviour was not restricted in any way during the study. On Earth, participants could choose to go to sleep and wake up anytime. During spaceflight, participants maintained a 24‐hr schedule based on Moscow time. There were no major schedule‐induced changes to sleep during the study (participant schedules are shown in Figures 1 and S1). The study was approved by the Beth Israel Deaconess Medical Center (2018P000786).

### 2.2 Objective sleep measurement

Participants were trained to self‐apply the “Nightcap” sleep monitor. Data collection was planned for three, 12‐night blocks preflight and inflight, with two, 12‐night blocks postflight. However, mission activities constrained the ability of the crewmembers to collect data at all of these time points. As a result, modifications to the data collection plan were required, resulting in fewer data collection points at variable times between participants. The actual timings of the data collection bouts by participant are included in Table S1.

The Nightcap is a validated device used to discriminate between REM, NREM and wake (Ajilore et al., 1995; Atienza et al., 2004; Cantero et al., 2002; Pace‐Schott et al., 1994; Silvestri et al., 2001). It has been validated against polysomnography, and demonstrates 85%–87% agreement between 1‐min sleep stage epochs (Ajilore et al., 1995; Cantero et al., 2002). The equipment consists of a motion detector attached to the head to monitor body movements, and a piezoelectric sticker attached to the left eyelid to measure eye movements.

### 2.3 Sleep staging analysis

The Nightcap counts eye and body movements for each minute, and the count totals are used to score recorded time as REM, NREM or wakefulness. For the present dataset, two researchers, blinded to subject identity, study phase and recording block, visualized and hand‐scored each minute as wakefulness, NREM or REM using a data visualization software called NightViewAM, developed in the Center for Sleep and Cognition. Specifically, the 1‐min intervals of the recordings that were characterized by both body and eye movements were scored as wakefulness, those marked by scarce eye and body movements were scored as NREM, and those with primarily eye movements alone were scored as REM, as shown in Figure S2.

We measured several variables from each night, including sleep opportunity, total sleep time (TST), sleep efficiency, sleep onset latency (SOL), wake after sleep onset (WASO), REM latency, total REM duration and percent of REM, and total NREM duration and percent of NREM. Participants were instructed to don the equipment and begin the recording when they were ready to sleep. Therefore, we considered “bedtime” as the clock time at which the subject initiated each recording, and “risetime” as the clock time at which the subject switched the Nightcap off again. “Sleep opportunity” represents the time between bedtime and risetime. From each scored night, we totaled NREM and REM time to calculate TST. Sleep efficiency was defined as the proportion of sleep opportunity spent sleeping. SOL was considered the time from bedtime until the first 5 min of consecutive sleep or as determined by the expert scorer. WASO was calculated as all the wake time after sleep onset but before risetime. REM latency was calculated as the number of minutes from the sleep onset until the first minute of scored REM sleep.

### 2.4 Data analysis

We computed descriptive statistics for all variables and study phases. All descriptive statistics are presented as the mean ± standard deviation unless otherwise indicated. Continuous sleep outcomes were compared between study phases using mixed‐effects regression models with participant and intercept as random effects (“proc mixed” in SAS). Percent changes were compared between study phases using mixed‐effects models that specified a beta distribution (“proc glimmix”). Our comparisons between spaceflight and each of the Earth‐based measures (preflight and postflight) yielded similar results. For simplicity, comparisons are presented relative to preflight. We report the estimated *β* coefficient for each of these models, which represents the predicted change in *y* for a unit change in *x*, holding other covariates constant (i.e. the difference between the outcome during spaceflight [1], compared with preflight [0]).

We estimated longitudinal changes in sleep outcomes over time in space using separate beta‐distributed generalized estimating equation (GEE) regression models to address the repeated measures within crew members over time (“proc glimmix”). We chose mixed models to account for the differences in timing between data collection bouts and between individuals. We chose GEE models over subject‐specific random intercept mixed models for longitudinal analysis because GEE models are more robust and result in more stable estimates of the mean population trend. Robust standard errors addressed non‐homogenous variance over time. Linear and natural cubic splines models by mission day were used to assess the inflight changes over time. Where linear response explained the data as well as the splines model, we chose the simpler model. Where a parameter was clearly non‐linear across time, we used splines to describe the entire time course. We specified a gamma distribution for skewed data (SOL, REM latency).

Finally, we calculated effect sizes for all comparisons using Hedges' *g* (denoted as *g* in all results), adjusted to reduce sample size bias as follows:

$$g=\frac{M_{1}-M_{2}}{{\mathrm{SD}}_{\text{pooled}}^{*}}\times \left(\frac{N-3}{N-2.25}\right)\times \sqrt{\frac{N-2}{N}}$$

### 2.5 Data exclusion

There were 68 nights of data excluded: 54 recordings for poor Nightcap signal quality, two for the recorded night being shorter than 2 hr, eight for the participant stopping the recording and continuing to sleep, and four for an outlying time after the final awakening. Although we set the minimum recording threshold to 2 hr, the final dataset did not include any recordings that were shorter than 4 hr.

## 3 RESULTS

Eight crew members agreed to participate in the study. All participants completed baseline data collection, but we excluded data from three participants who only served in backup roles for the crew and did not complete any spaceflight recordings. The remaining five were males with an average age of 43.5 years (± 3.8 years) at the time of launch. Participants spent an average of 180 (132–198 [range]) days living on the Russian space station, Mir, during two missions between 1996 and 1998. Altogether, the subjects collected 324 nights of sleep, of which 256 nights met our inclusion criteria (79% data yield). Participants provided an average of 22.4 usable preflight nights (including 32, 22, 21, 18, 19 nights for the five participants), 16.6 usable spaceflight nights (including 22, 7, 32, 18, 4 nights for the five participants, respectively) and 12.2 usable postflight nights (including 20, 10, 19, 5, 7 nights for the five participants, respectively) for a total of 112 nights preflight, 83 nights inflight and 61 nights postflight. A raster plot from one participant is shown in Figure 1. Raster plots showing the exact data collection times for the other four participants are included in Figure S1.

**FIGURE 2:**
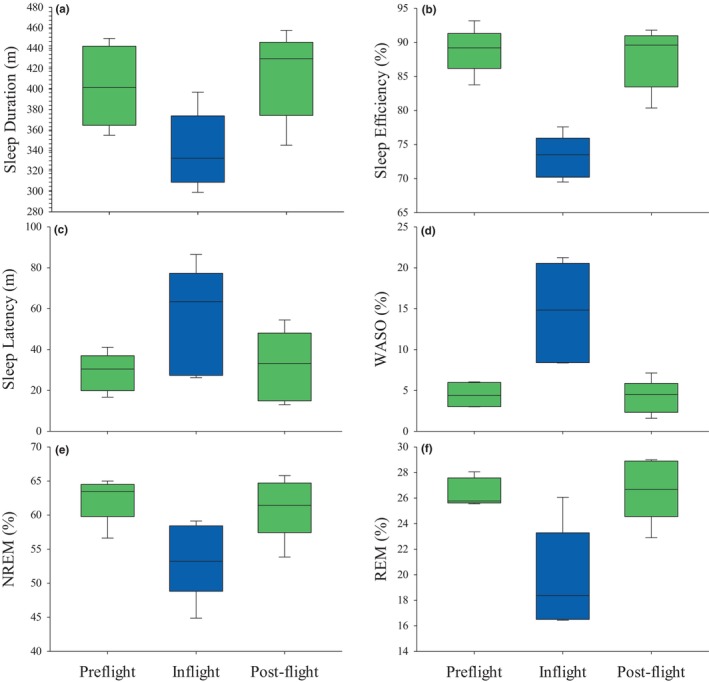
Overall changes in sleep outcomes by study phase. The upper and lower edges of each box represent the 25th and 75th percentiles, the line within the box represents the median, and the whiskers indicate the 10th and 90th percentiles. m, minutes; NREM, non‐rapid eye movement; REM, rapid eye movement; TST, total sleep time; WASO, wake after sleep onset; REM (%), NREM (%) = percent of TST. Green shading represents Earth‐based data collection, blue shading represents spaceflight data collection.

Confirming the findings of other studies, we found that participants slept significantly less in space compared with on Earth (5.7 ± 0.6 hr [mean ± s.e.m.] inflight versus 6.7 ± 0.7 hr preflight; *β* = −63.4, *p* = 0.0002, *g =* −0.93; Figure 2a) despite having the same sleep opportunity between study phases (7.6 ± 0.7 hr preflight versus 7.7 ± 0.6 hr inflight; *β* = 10.5, *p =* 0.29). On average, the crew members had a ~16% lower sleep efficiency inflight (73% ± 3% inflight versus 89% ± 3% preflight; *β* = −1.07, *p* = 0.0001, *g =* −3.00; Figure 2b). The average SOL during the missions (54.5 ± 26.3 min) was nearly double that of preflight (28.8 ± 9.4 min; *β* = 25.7, *p* = 0.002, *g =* 1.15; Figure 2c). There was also a significant increase in the percentage of WASO, which was 4.5% ± 1.5% (20 ± 8 min) during preflight, but more than tripled during spaceflight (14.5% ± 6.0%; *β* = 1.3, *p* = 0.001, *g =* 2.26; 67 ± 28 min; Figure 2d). The means and standard deviations for all outcomes extracted from the Nightcap by study phase are shown in Table S1.

The significant increase in wakefulness during spaceflight came at the expense of both REM and NREM sleep (Figure 2e,f). Compared with the preflight data collection, the percentage of sleep opportunity spent in REM decreased from 26.4% (± 1.1%) to 19.6% (± 4.1%; *β* = −0.4, *p* = 0.003, *g =* −1.82), and NREM decreased from 62.4% (± 3.3%) to 53.5% (± 5.6%; *β* = −0.4, *p* = 0.0001, *g =* −1.49). The number of minutes of REM (90 ± 14 min inflight) and NREM (250 ± 42 min inflight) was also significantly reduced compared with preflight (REM = 120 ± 9 min; *β* = −29.8, *p* = 0.002, *g =* −2.25; NREM = 283 ± 33 min; *β* = −33.5, *p* = 0.0002, *g =* −0.77). Surprisingly, REM latency decreased by nearly 30 min compared with preflight (91 ± 36 min versus 62 ± 10 min; *β* = 29.1, *p* = 0.047, *g =* 0.97).

There were no differences in any sleep outcomes between the preflight and postflight data collection phases, suggesting that spaceflight does not result in irreversible changes in the sleep metrics that we measured (Figure 2).

Over the course of time spent in space, sleep opportunity increased and then decreased significantly (splines model, *F* = 9.01, *p* = 0.01), but this did not result in significantly longer TST (linear model, *β* = 0.1, *p* = 0.73) because SOL lengthened significantly over time in space (linear model, *β* = 0.6, *p* = 0.003). This led to a significant reduction in sleep efficiency over time (splines model, *F* = 9.18, *p* = 0.009) despite WASO remaining unchanged with time in space (splines model, *F* = 0.36, *p* = 0.71).

**FIGURE 3:**
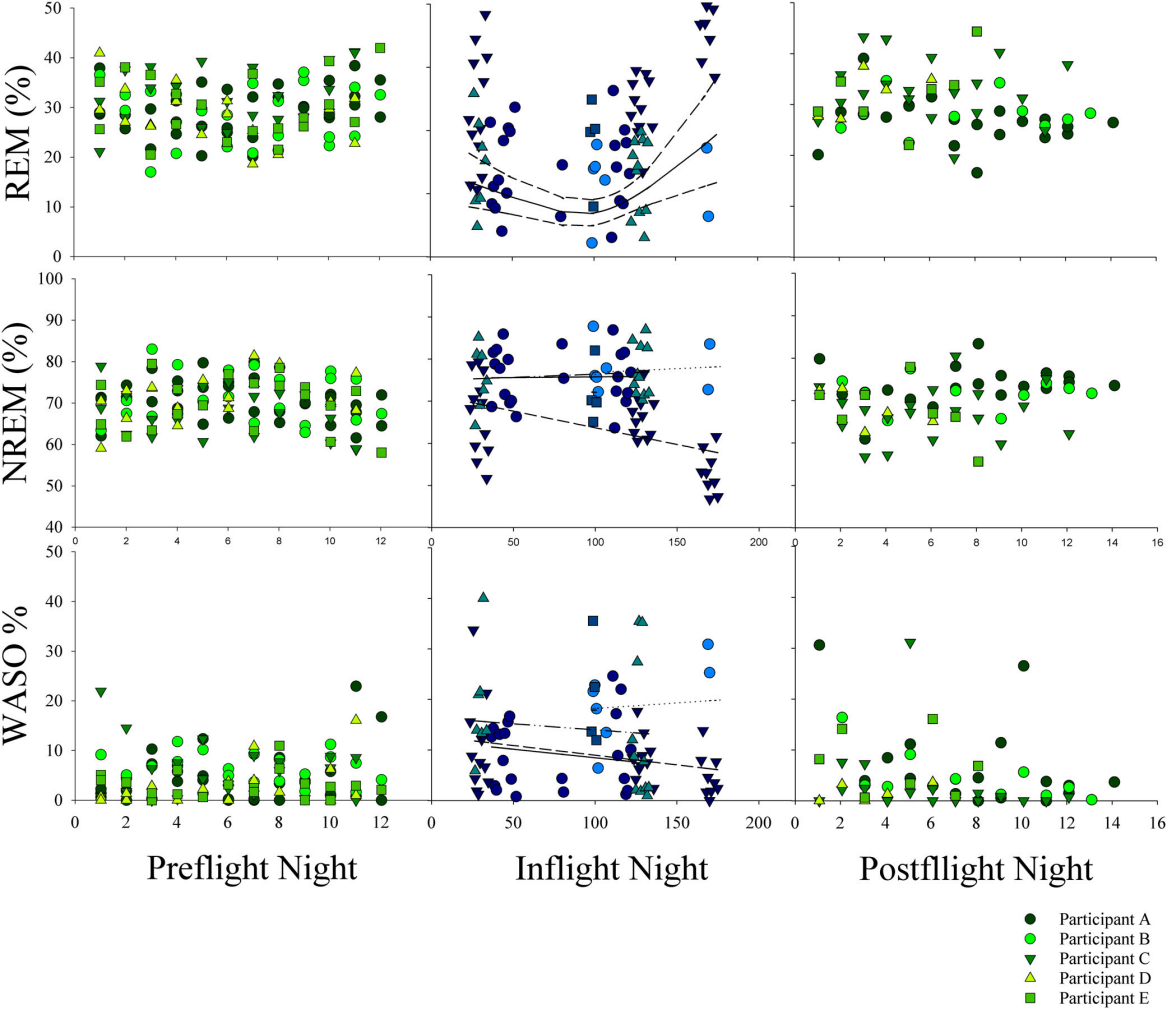
Changes in sleep outcomes over time by study phase. Each symbol represents a single participant (corresponding in blue and green). The pre‐ and postflight data collection bouts (two bouts of ~12 nights) are overlayed. Predicted splines (solid lines) and 95% confidence intervals (dashed lines) from the generalized estimating equation (GEE) models are plotted for the inflight data collection phase. Green shading represents Earth‐based data collection, blue shading represents spaceflight data collection. NREM, non‐REM sleep; REM, rapid eye movement sleep; WASO, wake after sleep onset.

The percentage of time spent in REM initially decreased and then increased significantly over time in space, returning to near preflight levels by ~180 days in space (*F* = 5.73, *p* = 0.03; Figure 3). REM latency followed an inverse pattern, with shorter REM latencies occurring when the percentage of REM was greater (*F* = 21.55, *p* < 0.001; Figure 3). The changes in REM sleep over time were accounted for by changes in NREM sleep (*F* = 17.45, *p* = 0.001; Figure 3).

## 4 DISCUSSION

Our study examined sleep architecture in the largest sleep data set collected during spaceflight to date. We confirm that average sleep duration is shorter in space compared with on Earth (over extended durations in space). We also demonstrate that sleep duration did not increase over the course of a long‐duration spaceflight mission. This shortened sleep duration appears to result from increased wake during the sleep episode and was accompanied by reductions in both REM and NREM sleep. However, over the course of time in space, REM sleep recovered to baseline levels at the expense of a further decrease in NREM.

Numerous studies have demonstrated that sleep duration is shorter in space compared with on Earth, with crew averaging approximately 6 hr per night inflight (Barger et al., 2014; Dijk et al., 2001; Frost Jr. et al., 1976; Gundel et al., 1997; Monk et al., 2001; Santy et al., 1988). Our findings are consistent with these reports and suggest that the reduced sleep duration seen in space is not simply due to a reduced opportunity for sleep but rather because of an increase in wakefulness during the sleep episode. Of note, we found that sleep opportunity increased with more time in space while sleep duration remained unchanged, owing to longer sleep latencies and increased WASO. It is unclear why the crew experienced longer sleep latency and WASO, but some conditions of the Mir missions, including environmental factors, stress and circadian misalignment might have contributed to these changes. For example, the Mir space station offered private sleep stations for crew members, but some reports have suggested that these sleep stations had light and noise pollution and poor temperature regulation, potentially leading to sleep disruption (Imhof et al., 2010). Stressful events during the Mir missions, including a fire and a near collision with a resupply vessel, may have provoked insomnia (Ellis, 2000). Circadian misalignment might have also contributed to the reduced sleep duration. Prior studies have argued that schedule‐induced circadian misalignment is responsible for reduced sleep duration during spaceflight (Flynn‐Evans, Barger, et al., 2016). Similarly, a recent study on the ISS found that crew members slept longer on weekends compared with weekdays (Jones et al., 2022), which suggests that crewmembers may experience abnormal circadian entrainment in space, as occurs with social jet lag on Earth (Wittmann et al., 2006). Although Mir operations maintained a 24‐hr schedule aligned with that of Earth‐based mission control, some reports from studies in spaceflight (Gundel et al., 1997; Monk et al., 2001; Stoilova et al., 2003) and analogue missions in ground‐based habitats or Antarctic expeditions (Rahman et al., 2022; Sletten et al., 2022) suggest that the circadian rhythms of crew members drifted later with time spent in mission, potentially due to lighting that was insufficient to maintain circadian entrainment during the crew's wake episodes. Such circadian drift cannot account for increased WASO inflight, but would explain the longer sleep latency that we observed over time in the mission. More specifically, as the circadian drive for wakefulness shifted later over the course of the mission, so too would the wake maintenance zone have been delayed, inhibiting sleep at the beginning of the crew's scheduled sleep opportunity and, thereby, increasing sleep latency; this occurs in 10%–20% of sleep‐onset insomnia cases (Flynn‐Evans et al., 2017). Additional studies with objective circadian phase markers collected simultaneously with objective sleep measures are needed to confirm the role of circadian phase in the development of spaceflight insomnia.

We found that both REM and NREM sleep were reduced in space compared with on Earth while wakefulness increased. This finding is consistent with short‐duration, Earth‐based laboratory (Skorucak et al., 2018; Van Dongen et al., 2003) and field (Cvirn et al., 2017) studies that demonstrate that chronic sleep restriction is associated with a reduction in both REM and NREM sleep. Such sleep losses are associated with neurobehavioural performance impairment on Earth (Van Dongen et al., 2003) and in space (Jones et al., 2022), highlighting the importance of identifying countermeasures to mitigate sleep loss during spaceflight. Hypnotics do not appear to be effective for increasing sleep duration during spaceflight (Barger et al., 2014), and this necessitates the exploration of other countermeasures that facilitate longer sleep duration (e.g., longer sleep opportunities, improved sleep environment), stabilize circadian entrainment (e.g. lighting or scheduling countermeasures), or enhance waking performance during sleep deficiency.

The reduction in sleep duration that we observed was accompanied by other changes in sleep architecture. Our finding that REM latency was longer during spaceflight compared with on Earth conflicts with prior reports that found a shorter REM latency inflight (Frost Jr. et al., 1976; Gundel et al., 1993; Gundel et al., 1997); differences in mission conditions, schedules and circadian misalignment may have been responsible for this discordance. Still, it is unclear why REM latency would be altered during spaceflight. Shorter REM latency is associated with depression (Riemann et al., 2020), while longer REM latency has been tied to endurance exercise (Driver & Taylor, 2000; Stutz et al., 2019). It is possible that the changes in REM latency that we observed could correlate with behavioural health changes and/or the physical exertion required to live in a microgravity environment (Fraser et al., 2012). The mechanisms by which these changes occur on Earth remain unclear, making it difficult to interpret this finding in the context of spaceflight.

Our finding that REM sleep recovered to baseline levels over the course of 6 months of chronic sleep loss may reflect a partial adaptation to the spaceflight environment or provide evidence for long‐term homeostatic REM regulation (Franken, 2002; Ocampo‐Garces et al., 2020). Additional studies are needed on Earth to determine whether long‐term chronic sleep reduction is associated with a similar process. Notably, as TST did not increase over the course of the mission, the increase in REM that we observed came at the expense of NREM sleep. As the device that we used for data collection could not discriminate between NREM substages, it is worth further investigating the aspects of NREM sleep that are affected. Earth‐based studies consistently find that chronic sleep restriction involves the preservation of SWS while stage 2 NREM sleep is reduced (Elmenhorst et al., 2008; Van Dongen et al., 2003). This could be a significant concern for future spaceflight missions because sleep spindles, which dominate stage 2 NREM sleep, are associated with learning (Peyrache & Seibt, 2020). A study examining sleep spindles during a short‐duration spaceflight found that fast spindle density and slow spindle frequency increased, suggesting that changes in spindle activity may facilitate adaptation to spaceflight, potentially for motor skill learning (Koller et al., 2021). It is unclear what impact, if any, the reduction in stage 2 NREM sleep and subsequent reductions in spindle activity may have over the course of a long‐duration spaceflight mission.

Although Earth‐based studies suggest that stage 2 NREM sleep would likely be sacrificed to preserve SWS and REM sleep, it is possible that the reduction in NREM sleep during long‐duration spaceflight follows a loss of SWS. Prior studies in space have reported that SWS is reduced relative to sleep on Earth during short‐duration missions (Koller et al., 2021; Monk et al., 1998), potentially due to fluid shifts in microgravity that affect the glymphatic system (Marshall‐Goebel et al., 2019). Further support that SWS may be affected during spaceflight comes from studies that show global increases in theta power and local sleep‐like events during waking (Petit et al., 2019), which are signatures of increased sleep pressure (Finelli et al., 2000). If SWS is reduced because of the conditions of spaceflight or displaced by REM recovery over long‐term chronic sleep loss, there may be significant consequences for those who live and work in space for long periods; for example, such sleep losses have been correlated with an increased risk of dementia (Nedergaard & Goldman, 2020). Given that losses of SWS and stage 2 NREM are both consequential and that we found prolonged deficits in NREM sleep during an average of 6 months of spaceflight, it is important to prioritize future electroencephalogram (EEG) studies to understand if and how spaceflight affects NREM sleep architecture.

While our findings are intriguing and significant, our study is not without limitations. The Nightcap's ability to differentiate between REM, NREM and wake has been validated (Ajilore et al., 1995; Cantero et al., 2002), and though its ease of use allowed us to collect a large volume of sleep data, it does not have EEG sensors that would have enabled us to quantify changes in the substages of NREM sleep. In addition, the Nightcap relies on changes in eye and head movements to distinguish REM from NREM and waking. A prior case study showed that eye movements may change during sleep in space (Quadens & Green, 1984). It is unclear how such changes may have influenced the algorithm that we used to classify sleep. We also had no information on accelerations or decelerations, which have been shown to affect EEG recordings (de Metz et al., 1992). Although the participants were instructed to don the equipment and begin the recordings only when they were ready to go to sleep, we cannot preclude the possibility that some crewmembers engaged in other activities on some nights before attempting to sleep. However, due to the inconvenience of wearing the sensors and recording equipment, we do not expect that this would have been a common or systematic occurrence. Another experimental shortcoming is with regards to the sample size and generalizability of our analyses: like most other studies examining sleep in space, the present study only includes data from a small pool of subjects representing relatively few demographic characteristics. When these data were collected, relatively few individuals lived and worked in space and there were limited options for assessing sleep architecture in a spaceflight environment. It is also unclear whether the individuals who participated in our study were normal sleepers. We found that the astronauts averaged 6.7 hr of sleep preflight and 6.9 hr of sleep postflight, which is less than self‐reported average sleep durations among the general population from that timeframe (Ford et al., 2015). The participants also had variable contributions of data during each phase of the study, and some only contributed a few recordings to the analysis for a given phase. The technological advancements in sleep recording that have occurred over the last 30 years should enable researchers to conduct studies in larger cohorts in the future. As a result of the small sample size, we used GEE models to evaluate longitudinal trends. However, GEE is not able to incorporate random slope effects per individual. With more subjects, we would be able to adjust our statistical methods and evaluate whether incorporating random slope effects results in better predictions of individual's responses. When we explored random intercept deviations with the current dataset, the models became unidentifiable and did not converge. We also do not have information on the participants' workload, detailed sleep environment information, or stress levels. Prior studies have shown that each of these factors can cause inflight sleep disruption (Barger et al., 2014; Jones et al., 2022). Furthermore, all participants were rigorously selected and highly trained professionals, which may have inadvertently selected for confounding traits in a homogenous way. As space tourism continues to become more common, more studies will be needed to determine whether individuals from the general population experience comparable changes in sleep duration and staging. Finally, we cannot disentangle the effects of Mir's sleep environment, potential circadian misalignment and workload on sleep. To determine whether spaceflight alone is responsible for the sleep changes that we observed, future studies should be conducted in an optimized sleep environment with sufficient lighting to facilitate circadian entrainment, a moderate workload and stable sleep–wake schedules.

In conclusion, we confirm that sleep duration is shorter during spaceflight compared with on Earth, and find that this shorter duration is accompanied by reductions in both REM and NREM sleep. These REM sleep deficits recovered over the course of an average of 6 months in space, at the expense of a further reduction in NREM. Overall, our findings support the need for further characterization of the long‐run recovery dynamics of REM sleep during prolonged chronic sleep restriction, examination of sleep during spaceflight, and generation of countermeasures to help future space visitors obtain sleep of adequate quality and quantity to support waking performance and long‐term health.

## AUTHOR CONTRIBUTIONS

**Oliver Piltch:** Formal analysis; writing – original draft; writing – review and editing. **Erin E. Flynn‐Evans:** Formal analysis; writing – original draft; writing – review and editing; supervision; validation. **Millennia Young:** Supervision; formal analysis. **Robert Stickgold:** Conceptualization; investigation; software; formal analysis; funding acquisition; project administration; writing – original draft; methodology; supervision; writing – review and editing.

## FUNDING INFORMATION

This study was funded by NAS 9‐19406, NIMH #MH‐48,832, the MacArthur Foundation Mind–Body Network, and Healthdyne Technologies, and Mr Piltch was funded by the Mary Gordon Roberts Fellowship. Drs Flynn‐Evans and Young were supported by the NASA Human Research Program.

## CONFLICT OF INTEREST STATEMENT

Mr Piltch and Dr Stickgold report no conflict of interest with this work. Drs Flynn‐Evans and Young are employed by NASA, who provided support for this study.

## Supporting information


**FIGURE S1.** Raster plots for four participants (participant 5 is shown in Figure 1). Numbers preceded by L− represent the night of preflight data collection relative to launch (L = launch). Numbers preceded by L+ represent inflight data collection nights after launch. Numbers preceded by R+ represent data collection during the postflight phase relative to return (R = return). Boxes represent sleep opportunity, white = in bed awake, dark grey = rapid eye movement (REM) sleep, light grey = non‐REM sleep (NREM), black = unscorable.

**FIGURE S2.** Nightcap Data Visualization. The top tracing within each panel shows eye movement counts, while the bottom charts body movements. The line between the tracing denotes the stage of sleep and exists at three heights. The highest level represents wakefulness, the intermediate, rapid eye movement (REM), and the bottom level signifies non‐REM sleep (NREM). These sample Nightcap recordings, all from the same participant, are displayed in NightViewAM, and highlight some of the differences between scorable and unscorable recordings. (a) The only scorable recording of the four with clear data from both sensors. (b) Unscorable recording with noisy data from both sensors. (c) Unscorable recording with clear body sensor data but noisy eye movements. (d) Unscorable recording with clear body movement counts but no eye movement counts.

**TABLE S1.** All sleep outcomes extracted from the Nightcap preflight, inflight and postflight.

## Data Availability

The deidentified datasets used in this analysis have been deposited in the NASA Life Sciences Data Archive (https://nlsp.nasa.gov/explore/lsdahome) and are publicly available upon request. The code used in this dataset has been deposited in the NASA Life Sciences Data Archive (https://nlsp.nasa.gov/explore/lsdahome) and is publicly available upon request. Any additional information required to reanalyse the data reported in this paper is available from the lead contact upon request.
