# Supplementary figures and images for: Changes to human sleep architecture during long‐duration spaceflight
Source: J Sleep Res. 2024 Nov 10;34(3):e14345. doi: 10.1111/jsr.14345 (PMC12069747; doi:10.1111/jsr.14345)

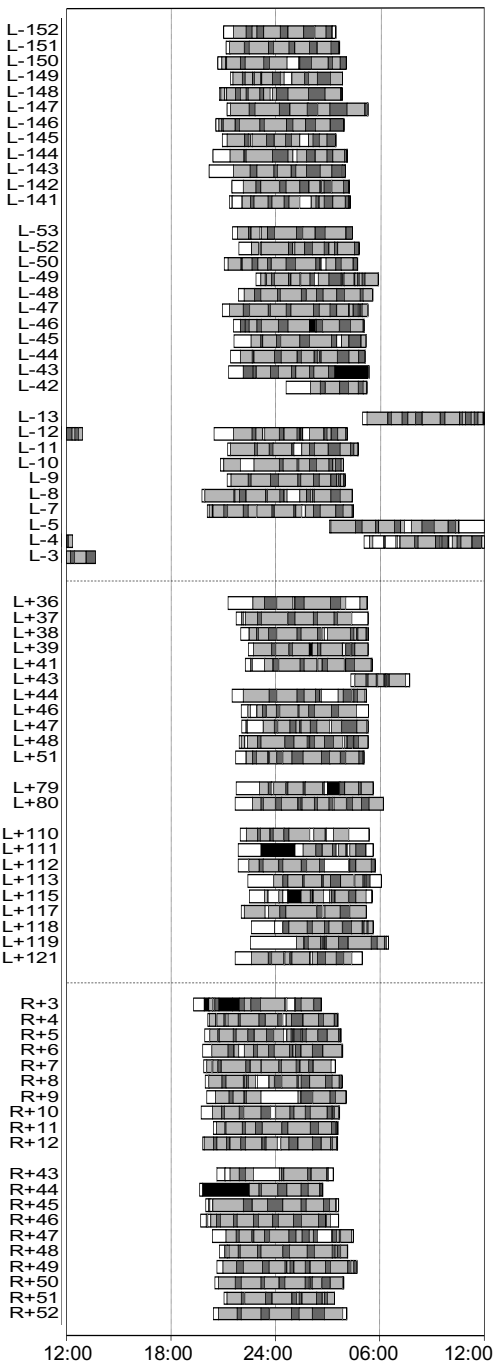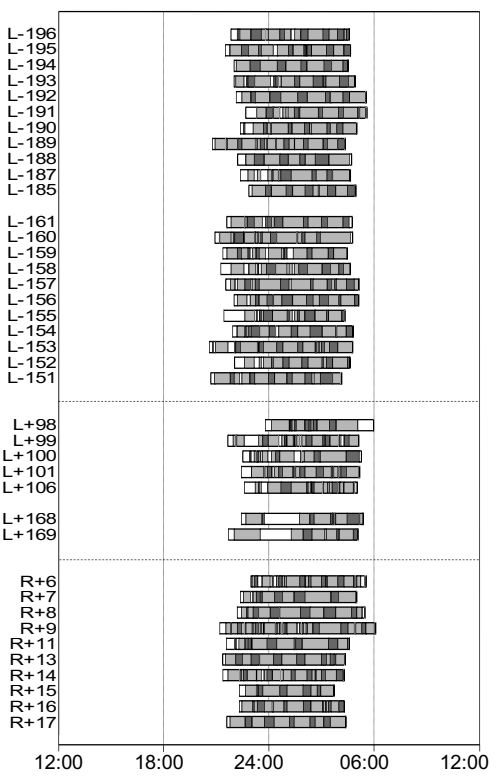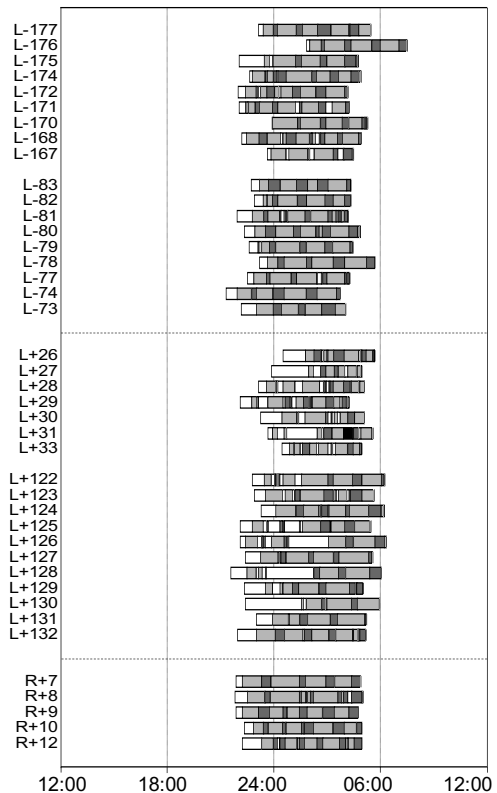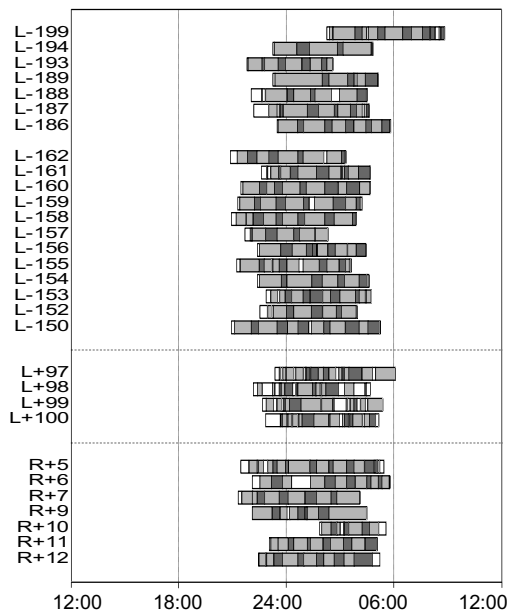

Supplement: Supplementary file 1 — FIGURE S1. Raster plots for four participants (participant 5 is shown in Figure 1). Numbers preceded by L− represent the night of preflight data collection relative to launch (L = launch). Numbers preceded by L+ represent inflight data collection nights after launch. Numbers preceded by R+ represent data collection during the postflight phase relative to return (R = return). Boxes represent sleep opportunity, white = in bed awake, dark grey = rapid eye movement (REM) sleep, light grey = non‐REM sleep (NREM), black = unscorable. [file JSR-34-e14345-s001.pdf]

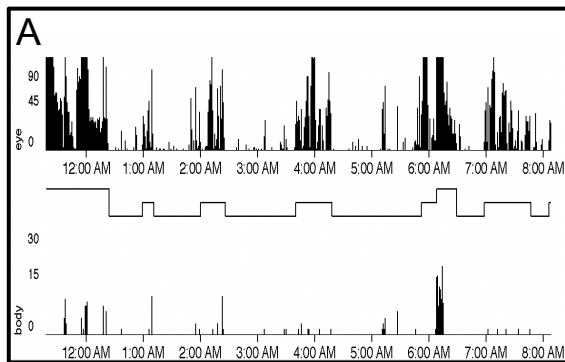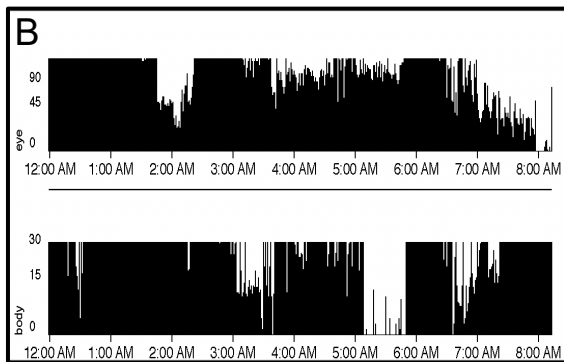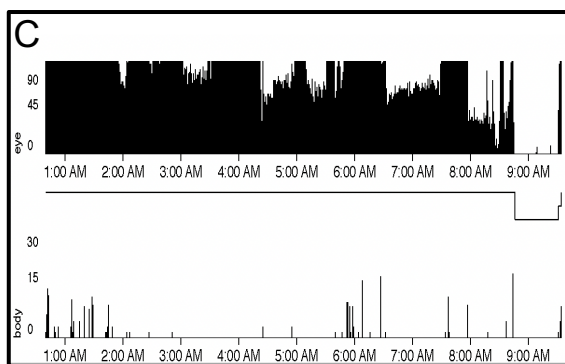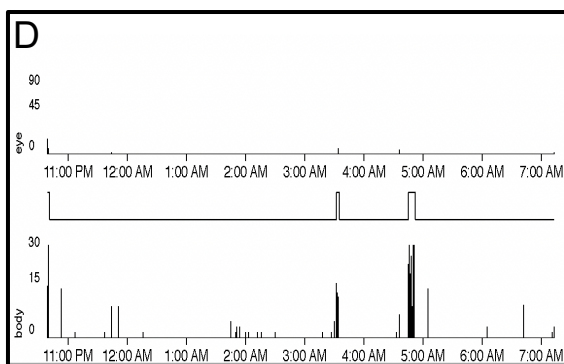

Supplement: Supplementary file 2 — FIGURE S2. Nightcap Data Visualization. The top tracing within each panel shows eye movement counts, while the bottom charts body movements. The line between the tracing denotes the stage of sleep and exists at three heights. The highest level represents wakefulness, the intermediate, rapid eye movement (REM), and the bottom level signifies non‐REM sleep (NREM). These sample Nightcap recordings, all from the same participant, are displayed in NightViewAM, and highlight some of the differences between scorable and unscorable recordings. (a) The only scorable recording of the four with clear data from both sensors. (b) Unscorable recording with noisy data from both sensors. (c) Unscorable recording with clear body sensor data but noisy eye movements. (d) Unscorable recording with clear body movement counts but no eye movement counts. [file JSR-34-e14345-s003.pdf]
