# Supplementary material for: Changes to human sleep architecture during long‐duration spaceflight
Source: J Sleep Res. 2024 Nov 10;34(3):e14345. doi: 10.1111/jsr.14345 (PMC12069747; doi:10.1111/jsr.14345)
Supplement: Supplementary file 3 — TABLE S1. All sleep outcomes extracted from the Nightcap preflight, inflight and postflight. [file JSR-34-e14345-s002.docx]

| **Supplemental Table 1.**  All sleep outcomes extracted from the Nightcap preflight, inflight, and postflight. | | | |
| --- | --- | --- | --- |
| **Variable** | **Preflight (N = 112)** | **Spaceflight (N = 83)** | **Postflight (N = 61)** |
| Sleep Opportunity, min | 453.8 ± 40.5 | 464.3 ± 34.0 | 471.7 ± 38.1 |
| Total Sleep Time, min | 403.0 ± 39.7 | 339.6 ± 37.3 | 414.0 ± 43.0 |
| Sleep Efficiency, % | 88.8 ± 3.3 | 73.1 ± 3.1 | 87.6 ± 4.5 |
| REM, min | 120.0 ± 8.7 | 90.1 ± 14.2 | 125.0 ± 8.6 |
| REM, % sleep time | 29.8 ± 1.6 | 27.0 ± 6.1 | 30.4 ± 2.8 |
| REM, % total recording | 26.4 ± 1.1 | 19.6 ± 4.1 | 26.6 ± 2.5 |
| NREM, min | 283.0 ± 32.5 | 249.5 ± 41.7 | 289.0 ± 39.9 |
| NREM, % sleep time | 70.2 ± 1.6 | 73.0 ± 6.1 | 69.6 ± 2.8 |
| NREM, % total recording | 62.3 ± 3.3 | 53.5 ± 5.6 | 61.0 ± 4.5 |
| WASO, min | 20.4 ± 7.7 | 67.1 ± 28.5 | 19.8 ± 9.1 |
| WASO, % total recording | 4.5 ± 1.5 | 14.4 ± 6.0 | 4.2 ± 2.1 |
| Wakefulness, min | 49.2 ± 13.3 | 121.7 ± 11.1 | 51.6 ± 11.4 |
| Wakefulness, % total recording | 11.0 ± 3.1 | 26.3 ± 3.7 | 11.1 ± 3.0 |
| Nonscorable, min | 1.5 ± 2.3 | 2.7 ± 4.9 | 6.0 ± 8.2 |
| Nonscorable, % total recording | 0.3 ± 0.4 | 0.5 ± 0.9 | 1.3 ± 1.7 |
| Sleep Latency, min | 28.8 ± 9.4 | 54.5 ± 26.3 | 31.8 ± 17.3 |
| REM Latency, min | 62.4 ± 10.0 | 91.5 ± 35.5 | 55.0 ± 6.2 |
| Bedtime | 00:15 ± 00:37 | 00:49 ± 00:39 | 23:36 ± 01:13 |
| Risetime | 07:49 ± 00:12 | 08:33 ± 00:10 | 07:28 ± 00:44 |
